# Gastric Section Correlation Network for Gastric Precancerous Lesion Diagnosis

**DOI:** 10.1109/OJEMB.2023.3277219

**Published:** 2023-05-17

**Authors:** Jyun-Yao Jhang, Yu-Ching Tsai, Tzu-Chun Hsu, Chun-Rong Huang, Hsiu-Chi Cheng, Bor-Shyang Sheu

**Affiliations:** Department of Computer Science and EngineeringNational Chung Hsing University 34916 Taichung 402 Taiwan; Department of Internal MedicineTainan Hospital, Ministry of Health and Welfare 156935 Tainan 701 Taiwan; Department of Internal Medicine, National Cheng Kung University Hospital, College of MedicineNational Cheng Kung University 34912 Tainan 701 Taiwan; Cross College Elite Program, and Academy of Innovative Semiconductor and Sustainable ManufacturingNational Cheng Kung University 34912 Tainan 701 Taiwan; Department of Computer Science and EngineeringNational Chung Hsing University 34916 Taichung 402 Taiwan; Department of Internal Medicine, Institute of Clinical Medicine and Molecular MedicineNational Cheng Kung University 34912 Tainan 701 Taiwan; Department of Internal MedicineTainan Hospital, Ministry of Health and Welfare 156935 Tainan 701 Taiwan; Institute of Clinical Medicine and Department of Internal Medicine, National Cheng Kung University HospitalNational Cheng Kung University 34912 Tainan 701 Taiwan

**Keywords:** Corpus-predominant gastritis index, deep learning, precancerous lesion classification, gastric endoscopy

## Abstract

*Goal:* Diagnosing the corpus-predominant gastritis index (CGI) which is an early precancerous lesion in the stomach has been shown its effectiveness in identifying high gastric cancer risk patients for preventive healthcare. However, invasive biopsies and time-consuming pathological analysis are required for the CGI diagnosis. *Methods:* We propose a novel gastric section correlation network (GSCNet) for the CGI diagnosis from endoscopic images of three dominant gastric sections, the antrum, body and cardia. The proposed network consists of two dominant modules including the scaling feature fusion module and section correlation module. The front one aims to extract scaling fusion features which can effectively represent the mucosa under variant viewing angles and scale changes for each gastric section. The latter one aims to apply the medical prior knowledge with three section correlation losses to model the correlations of different gastric sections for the CGI diagnosis. *Results:* The proposed method outperforms competing deep learning methods and achieves high testing accuracy, sensitivity, and specificity of 0.957, 0.938 and 0.962, respectively. *Conclusions:* The proposed method is the first method to identify high gastric cancer risk patients with CGI from endoscopic images without invasive biopsies and time-consuming pathological analysis.

## I. Introduction

To Control the disease burden of the gastric cancer (GCA), it is important to diagnose the presence of the precancerous lesions in the stomach for the early detection of high GCA risk patients to reduce the mortality rate and occurrence of gastric cancer. Compared with gastric lesion classification methods [1], [2], [3], [4], [5], [6], [7], [8], [9] and segmentation methods [10], [11], [12], [13], [14], detecting precancerous lesions will help physicians achieve early detection of GCA in preventive healthcare. Recently, the corpus-predominant gastritis index (CGI) [15] is proposed to assess the precancerous lesions of high GCA risks and indicate candidates for early *Helicobacter pylori* (*H. pylori*) eradication before the presence of gastric intestinal metaplasia (IM). In the previous studies, CGI has been shown an early and reversible marker for the diagnosis of high GCA risks [16]. According to [15] and [16], CGI is a good marker because its presence could indicate high GCA risk early before the presence of atrophy and gastric IM in the previous researches [17], [18], [19]. CGI has also been shown an early and reversible marker for the diagnosis of high GCA risks [16]. Thus, the CGI diagnosis is important in the routine endoscopy study.

In order to diagnose CGI, the pathologists need to analyze biopsy specimens obtained from three gastric sections including the antrum, body and cardia, respectively. Then, they assort the inflammation scores of these three gastric sections by two indices, acute inflammation score (AIS) and chronic inflammation score (CIS) according to the updated Sydney system [20]. Both of AIS and CIS are ranged from 0 to 3 to represent the degrees of inflammations. The 0 score represents that the biopsy is normal, and the higher the number indicates more severe inflammations. After figuring out the scores of AIS and CIS, the pathologists combine these two indexes to an inflammation score (IS) which assesses the overall inflammations of the target gastric section. Based on the correlations of the inflammation scores of these three gastric sections, the CGI of each patient can be diagnosed as follows [15]:

\begin{equation*}
CGI = {\begin{cases}1,& \text{if } (IS_{A} < IS_{B})\\
 1, & \text{if } (IS_{A} \leq IS_{C} \ \text {and} \ IS_{C} \ne 1),\\
 0, &\text{otherwise} \end{cases}} \tag{1}
\end{equation*}

where $IS_{A}$, $IS_{B}$, and $IS_{C}$ are the inflammation scores of the antrum, body, and cardia, respectively. When the inflammation scores of the gastric sections of a patient match the first two criteria in (1), the patient has CGI, i.e. the patient is diagnosed as a high GCA risk patient.

This conventional process of the CGI presence in [15] relies on the topographic invasive biopsies and the protocol of the updated Sydney system [20]. It requires manual reviews of pathologists and is very time-consuming. Moreover, biopsies cause more bleeding risks of patients. As a result, a novel CGI diagnosis method without biopsies and the time-consuming burden of the pathologists becomes one of the most important and novel issues of detecting precancerous lesions for early GCA.

**Fig. 1.**
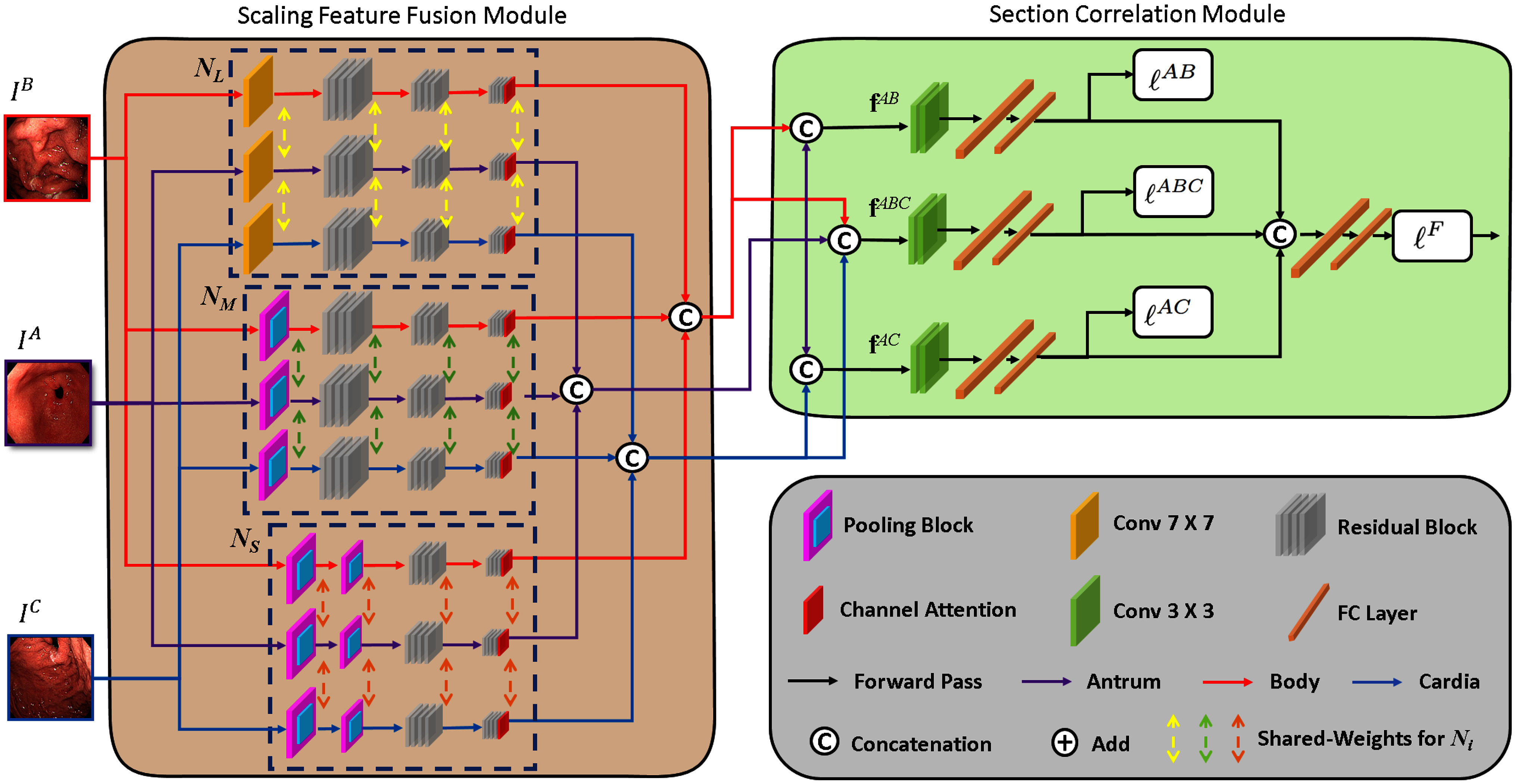
The overview of the proposed gastric section correlation network (GSCNet). The proposed network contains the scaling feature fusion module and section correlation module. While the front one aims to learn representative features for different gastric sections, the latter one considers the correlations of different gastric sections to achieve the CGI diagnosis. The dash rectangles represent the shared-weights resizing sub-networks in the scaling feature fusion module.

Recently, deep learning based computer-aided diagnosis (CAD) methods [21], [22], [23] have attracted more attention in the medical domain. Compared with previous CAD methods, we propose the first deep learning based CAD method to diagnose CGI from endoscopic images of the antrum, body and cardia, and aim to replace [15] which requires invasive biopsies. In our method, a novel deep learning based gastric section correlation network (GSCNet) is proposed. It consists of the scaling feature fusion (SFF) module and section correlation (SC) module as shown in Fig. 1. The endoscopic images of the antrum, body and cardia serve as the inputs of the network for the CGI diagnosis.

Due to the peristalsis of the stomach and different endoscopes, the distances and viewing angles between the mucosa of different patients and the cameras are variant and inconsistent. These situations lead to significant scale changes of the endoscopic images. To solve the problems, we propose the scaling feature fusion module, which contains three different shared-weights resizing sub-networks with different field-of-views. Each shared-weights resizing sub-network learns deep features which can simultaneously represent inflammations of three gastric sections with respect to the target scale. Moreover, we apply a channel attention layer [24] to each sub-network to extract more salient deep features. As a result, the learned features of the proposed method have better generalization ability and are more distinctive to represent different gastric sections. By concatenating the deep features of each shared-weights resizing sub-network, scaling fusion features of each gastric section are obtained and applied to learn the correlations of inflammations based on different gastric sections.

The medical prior knowledge to diagnose CGI represented by (1) in [15] shows that the correlations of inflammations of biopsy specimens of different gastric sections need to be considered to assess CGI of patients. In our approach, we consider the correlations of endoscopic images of different gastric sections for the CGI diagnosis and propose the section correlation module to represent the medical prior knowledge. This module aggregates scaling fusion features of the antrum with respect to those of the remaining gastric sections to represent the correlations of different gastric sections. Three section correlation losses are proposed and the features are fused to compute the fusion loss to drive the learning of the whole network. In this way, the proposed method can successfully represent the correlations of different gastric sections for the CGI diagnosis. As shown in the experimental results, the proposed method outperforms the state-of-the-art deep learning methods.

The contribution of the proposed method is three-fold:

1) First, this is the first artificial intelligence method to diagnose the precancerous lesions, CGI, from endoscopic images in an end-to-end trainable manner. We also reveal a novel medical application in video endoscopy to identify the high GCA risk patients.
2) Second, we propose a novel gastric section correlation network (GSCNet) which can learn representative deep features to overcome viewing direction and scale change problems, and correlate features of different gastric sections based on medical prior knowledge for the CGI diagnosis.
3) Third, our method significantly outperforms the state-of-the-art deep learning methods and shows the importance of imposing medical prior knowledge in the task-specific application.

## II. Materials and Methods

To solve the CGI diagnosis problem, we propose a novel gastric section correlation network (GSCNet) which consists of two dominant modules including the scaling feature fusion module and section correlation module as shown in Fig. 1. The scaling feature fusion module aims to extract representative scaling fusion features from endoscopic images of each gastric section. The section correlation module aims to represent the medical prior knowledge of CGI and provides interpretable network structure. Then, three section correlation losses are proposed and fused with the fusion loss to drive the network training and achieve the CGI diagnosis. In the following, we will describe the details of the proposed method.

### A. Scaling Feature Fusion Module

To solve the first problem and generate representative features for the CGI diagnosis, we propose the scaling feature fusion module to simultaneously extract gastric section features with respect to different scales and field-of-views for different gastric sections. Conventionally, to learn multi-scale features, most recent methods [25], [26] downsample input images to different scales in advance. These methods usually apply parallel multi-scale shared-weights networks with the fixed depth to learn multi-scale features from training images of a specific gastric section. Thus, the learned features easily overfit the training images of the specific gastric section, and are hard to simultaneously represent three endoscopic images of different gastric sections, i.e. the antrum, body, and cardia, which are required for the CGI diagnosis. Moreover, parallel multi-scale shared-weights networks of the same depth are hard to learn representative features with respect to different field-of-views.

Compared with the conventional multi-scale methods, the proposed scaling feature fusion module contains three individual shared-weights resizing sub-networks which can automatically achieve the resizing process by using the proposed network structure. It is not necessary to manually downsample the input images to obtain the inputs of different scales for the scaling feature fusion module in advance. Moreover, each sub-network simultaneously learns representative deep features from three endoscopic images of the antrum, body and cardia with respect to the target scale instead of images of a specific gastric section in most multi-scale methods. To further learn features of different field-of-view information and ensure the consistency of feature dimension for feature fusion, each sub-network also contains different numbers of convolutional layers and residual blocks. In this way, the learned features are more distinctive to represent different gastric sections under different scales and field-of-views.

Let $I^{A}$, $I^{B}$ and $I^{C}$ be the endoscopic images of a patient's antrum, body and cardia, respectively. These images serve as the inputs of the three individual shared-weights resizing sub-networks as shown in Fig. 1. The first shared-weights resizing sub-network ${N}_{L}$ contains a $7 \times 7$ convolutional layer and 3 residual blocks [27] to learn the large scale deep features from the input endoscopic images. Then, we append a channel attention layer [24] after the last residual block. The channel attention layer exploits the inter-channel relationship of features and helps further extract more salient deep features to represent each gastric section. By using the channel attention layer, the network can focus on learning critical content of different gastric sections. $N_{L}$ simultaneously generates large scale deep features $\mathbf {f}_{L}^{A}$, $\mathbf {f}_{L}^{B}$ and $\mathbf {f}_{L}^{C}$ from $I^{A}$, $I^{B}$ and $I^{C}$, respectively, instead of generating multi-scale features for only a specific gastric section in most conventional multi-scale methods. Because $N_{L}$ needs to learn features to simultaneously represent these three different gastric sections, the learned large scale deep features can be more distinctive and representative compared with conventional multi-scale methods.

The second shared-weights resizing sub-network ${N}_{M}$ aims to simultaneously learn features based on medium field-of-view information from $I^{A}$, $I^{B}$ and $I^{C}$. To achieve the goal, a pooling block, which is composed of a $3 \times 3$ convolutional layer with a max pooling layer, is applied to learn the medium scale information at first. After the pooling block, 3 residual blocks with a channel attention layer are applied in ${N}_{M}$ to generate the medium scale features $\mathbf {f}_{M}^{A}$, $\mathbf {f}_{M}^{B}$ and $\mathbf {f}_{M}^{C}$ computed from $I^{A}$, $I^{B}$ and $I^{C}$, respectively.

Finally, the third shared-weights resizing sub-network ${N}_{S}$ has 2 pooling blocks to obtain small scale information. Then, 2 residual blocks with a channel attention layer are applied to generate the small scale features $\mathbf {f}_{S}^{A}$, $\mathbf {f}_{S}^{B}$ and $\mathbf {f}_{S}^{C}$ for $I^{A}$, $I^{B}$ and $I^{C}$, respectively. In this way, the scaling feature fusion module can effectively learn distinctive and representative deep features for different gastric sections with respect to different scales and field-of-views by using three shared-weights resizing sub-networks of different depths and structures.

Because of the design of the network structure of $N_{L}$, $N_{M}$ and $N_{S}$, we can concatenate the deep features of different networks to integrate different scale and field-of-view information for each gastric section. Let $\mathbf {f}^{A}$ be the scaling fusion feature of the antrum. It is defined as the concatenation of deep features $\mathbf {f}^{A}_{L}$, $\mathbf {f}^{A}_{M}$ and $\mathbf {f}^{A}_{S}$ as follows:

\begin{equation*}
\mathbf {f}^{A} = \lbrace \mathbf {f}^{A}_{L}, \: \mathbf {f}^{A}_{M}, \: \mathbf {f}^{A}_{S}\rbrace. \tag{2}
\end{equation*}

Similarly, the scaling fusion feature $\mathbf {f}^{B}$ of the body is defined as:

\begin{equation*}
\mathbf {f}^{B} = \lbrace \mathbf {f}^{B}_{L}, \: \mathbf {f}^{B}_{M}, \: \mathbf {f}^{B}_{S}\rbrace, \tag{3}
\end{equation*}

and the scaling fusion feature $\mathbf {f}^{C}$ of the cardia is defined as:

\begin{equation*}
\mathbf {f}^{C} = \lbrace \mathbf {f}^{C}_{L}, \: \mathbf {f}^{C}_{M}, \: \mathbf {f}^{C}_{S}\rbrace. \tag{4}
\end{equation*}

In the proposed method, $\mathbf {f}^{A}$, $\mathbf {f}^{B}$, and $\mathbf {f}^{C}$ provide the different scale and field-of-view information of each gastric section by using shared-weights backbones.

Compared with conventional parallel multi-scale methods which learn different field-of-view information for the endoscopic images of each gastric section individually, the proposed SFF module aims to simultaneously learn representative deep features from endoscopic images of three different gastric sections, i.e. the antrum, body and cardia, with respect to the target scale. Each sub-network of the SFF module needs to learn the deep features which can simultaneously represent endoscopic images of different gastric sections based on different numbers of convolutional layers and residual blocks. In this way, the SFF module has better generalization ability and is able to reduce the overfitting problem. The deep features extracted from $N_{L}$ preserve more local details, while the deep features extracted from $N_{S}$ provide non-local information of the gastric sections in the following. By combining the different scale and field-of-view information with the weights-shared scheme, the learned deep features of the antrum, body and cardia can be more representative and discriminative. These features serve as the input of the section correlation module to achieve the CGI diagnosis.

### B. Section Correlation Module

As indicated in [15], the presence of CGI is defined as the occurrence of either: 1) the combination of acute inflammation and chronic inflammation scores in cardia (high corpus) is equal to or larger than that of the antrum, but is not equal to 1 in score; or 2) the combination of acute inflammation and chronic inflammation scores in the body (corpus) is larger than that of the antrum. Comparing body (corpus) and cardia (high corpus) is defined as an absence of CGI in [15]. In other words, only the correlations between the antrum and corpus including the body and cardia need to be considered based on the medical observations. Thus, we propose the section correlation module which combines scaling fusion features of different gastric sections and section correlation losses for the CGI diagnosis from endoscopic images. To address the correlations of different gastric sections, we combine scaling fusion features of different gastric sections to generate heterogeneous section features. The first heterogeneous section feature $\mathbf {f}^{AB}$ is obtained by the concatenation of the scaling fusion features of the antrum and body as follows:

\begin{equation*}
\mathbf {f}^{AB} = \lbrace \mathbf {f}^{A}, \: \mathbf {f}^{B}\rbrace. \tag{5}
\end{equation*}

It serves as the first feature of the correlations between the antrum and body for the CGI diagnosis. Then, $\mathbf {f}^{AB}$ is passed to two $3 \times 3$ convolutional layers to further update the correlations of the features between the antrum and body as shown in Fig. 1. The convolutional features are passed to two fully connected layers and a classification layer $c^{AB}$ to compute the first proposed section correlation loss $\ell ^{AB}$ which indicates the CGI diagnosis based on the correlation of the antrum and body as follows:

\begin{equation*}
\ell ^{AB} = \sum _{k=1}^{K} -y_{k} \log _{2} \left(p^{AB}_{k}\right), \tag{6}
\end{equation*}

where $K$ is the number of the training data, $y_{k}$ is the ground truth label provided in [15], and $p^{AB}_{k}$ is the output of $c^{AB}$ of the $k$th patient.

The second heterogeneous section feature $\mathbf {f}^{AC}$ aims to represent the correlations of inflammations between the antrum and cardia as follows:

\begin{equation*}
\mathbf {f}^{AC} = \lbrace \mathbf {f}^{A}, \: \mathbf {f}^{C}\rbrace. \tag{7}
\end{equation*}

Similar to the first heterogeneous section feature, $\mathbf {f}^{AC}$ also passes to a network of the same structure with a classification layer $c^{AC}$ to compute the second proposed section correlation loss $\ell ^{AC}$ which indicates the CGI diagnosis based on the antrum and cardia as follows:

\begin{equation*}
\ell ^{AC} = \sum _{k=1}^{K} -y_{k} \log _{2} \left(p^{AC}_{k}\right), \tag{8}
\end{equation*}

where $p^{AC}_{k}$ is the output of $c^{AC}$ of the $k$th patient. Based on these two heterogeneous section features and section correlation losses, we can represent the two medical criteria of the CGI diagnosis as shown in [15], where the first criterion represents the correlation between the antrum and body, and the second criterion represents the correlation between the antrum and cardia.

To further take the advantages of all of gastric sections for the CGI diagnosis, we further propose the third heterogeneous section feature $\mathbf {f}^{ABC}$ which is the concatenation of scaling fusion features of the antrum, body and cardia as follows:

\begin{equation*}
\mathbf {f}^{ABC} = \lbrace \mathbf {f}^{A}, \:\mathbf {f}^{B}, \: \mathbf {f}^{C}\rbrace. \tag{9}
\end{equation*}

$\mathbf {f}^{ABC}$ can be considered to discover the inflammation information based on the correlations among the antrum, body, and cardia for the CGI diagnosis. Based on $\mathbf {f}^{ABC}$ and the convolutional network mentioned above, the third proposed section correlation loss $\ell ^{ABC}$ is defined as follows:

\begin{equation*}
\ell ^{ABC} = \sum _{k=1}^{K} -y_{k} \log _{2} (p^{ABC}_{k}), \tag{10}
\end{equation*}

where $p^{ABC}_{k}$ is the output of the classification layer $c^{ABC}$ of the network. To show the effectiveness of the third section correlation loss $\ell ^{ABC}$, we perform the ablation study in Sec. IV-B.

To address the medical prior knowledge, these three losses are computed based on each heterogeneous section feature which represents the correlations of different gastric sections. Finally, to provide the fusion results of $c^{AB}$, $c^{AC}$ and $c^{ABC}$ for end-to-end training, we concatenate the features of these classifiers and pass to a classification layer $c^{F}$ to compute the fusion loss $\ell ^{F}$ as follows:

\begin{equation*}
\ell ^{F} = \sum _{k=1}^{K} -y_{k} \log _{2} \left(p^{F}_{k}\right), \tag{11}
\end{equation*}

where $p^{F}_{k}$ is the output of the classification layer $c^{F}$ of the network.

To update the parameters of the scaling feature fusion module and section correlation module by simultaneously considering all of the aforementioned losses, we compute the CGI loss $\ell$ as follows:

\begin{equation*}
\ell = \ell ^{AB} + \ell ^{AC} + \ell ^{ABC} + \ell ^{F}. \tag{12}
\end{equation*}

The CGI loss fuses these four losses and drives the learning of the proposed network in an end-to-end trainable manner. By minimizing the CGI loss, the learned scaling fusion features of each gastric section and heterogeneous section features of different gastric sections can then effectively and better represent the inflammations of the gastric mucosa for the CGI diagnosis. In this way, the proposed network can successfully solve the CGI diagnosis problem under variant scale changes. Moreover, because the design of the module is based on the medical prior knowledge, our method is more interpretable and convincing to physicians.

While most recent CAD methods aim to diagnose diseases from images containing target lesions, the CGI diagnosis requires to consider the correlations of inflammations between different gastric sections [15]. Thus, the CAD method for the CGI diagnosis also needs to simultaneously consider the medical prior knowledge mentioned above instead of only considering the content of each endoscopic image individually. The state-of-the-art CAD methods are then not suitable for the CGI diagnosis. Such fact shows the differences between the proposed method and the recent CAD methods, and also addresses the importance and necessary of developing a novel method for the CGI diagnosis.

### C. Implementation Details

In our implementation, the resolutions of the endoscopic images $I^{A}$, $I^{B}$ and $I^{C}$ are $224 \times 224$. The residual blocks used in the scaling feature fusion module are from the pre-trained ResNet-18 network [27], which can reduce the training time and avoid overfitting. Because the spatial resolution of the features of the last residual block in ResNet-18 are $7\times 7$, we remove the last block of ResNet-18 to obtain features with higher resolutions of $14 \times 14$. Then, we append the channel attention layer of the squeeze-and-excitation block [24] after the last residual block of each shared-weights resizing sub-network to make the learned features focus on critical parts of the input images of different gastric sections. In this way, the spatial resolution of each scaling fusion feature of each gastric section is $14 \times 14$ with 256 channels. During training, the batch size was set to 68 and the learning rate was $1e-5$. For the optimization strategy, we choose Adam [28] and train 1000 epochs. To prevent overfitting and increase the generalization ability of the learned features, we also apply data augmentations including random cropping, color jittering, grayscale, horizontal flip, vertical flip, and rotations of 90, 180 and 270 degrees during training.

## III. Results

### A. Dataset

In this study, patients older than 20 years old with the normal mental function who suffered from dyspepsia and received pan-endoscopy were invited from the National Cheng Kung University hospital and Ministry of Health and Welfare Tainan hospital. They received $H. pylori$ screening and endoscopy to provide topographic gastric biopsy specimens for histological assessment. Patients with the following conditions were excluded: 1) Patients with severe systemic diseases, such as severe anemia, uremia, liver cirrhosis with portal hypertensive gastropathy and malignancies except GCA; 2) Patients with bleeding tendency such as thrombocytopenia, long term NSAIDs or Aspirin treatment. The endoscopic images were collected topographically at the antrum, body and cardia with Olympus EVIS CV 290 system and H290 gastro-scope or EVIS CV 290 and Q290 gastroscope. The CGI of each patient was assessed by one experienced pathologist who was blind to the clinical information of the patients. Gastric mucosa biopsy specimens were reviewed and scored AIS and CIS according to the updated Sydney system [20]. The ground truth of the CGI diagnosis was provided by [15] based on the scored AIS and CIS. All of the patients were given written informed consent and the informed consent forms and study design were reviewed by the Research Ethics Committee of the institute.

The dataset contains 304 patients for the CGI diagnosis and each patient has 3 white-light endoscopic images of the antrum, body and cardia. The images of three gastric sections were selected and pre-processed by using [29]. The training and testing data were randomly partitioned as $7:3$ are shown in Table I. To avoid the overfitting and make the model more robust for rotations of endoscopic cameras, we performed data augmentations. Because no validation sets are provided, we trained the network with fixed epochs. Please refer to the implementation details. Our network was implemented by using Pytorch 1.7.1 and run on a personal computer with NVIDIA GeForce RTX 3090.

**TABLE I:** Dataset

# of Patients	With CGI	Without CGI	Total
Training	68	90	212
Testing	20	48	92

To evaluate the performance of the proposed method, the accuracy, sensitivity, specificity and area under receiver operating characteristic curve (AUC) are used. Let $TP$, $TN$, $FP$ and $FN$ be the numbers of true positive, true negative, false positive and false negative, respectively. The accuracy (Acc), sensitivity (Sens) and specificity (Spec) are defined as follows:

\begin{align*}
Accuracy =& \frac{TP+TN}{TP+TN+FP+FN}, \tag{13}
\\
Sensitivity =& \frac{TP}{TP+FN}, \tag{14}
\end{align*}

and

\begin{equation*}
Specificity = \frac{TN}{TN+FP}. \tag{15}
\end{equation*}

### B. Ablation Study

To diagnose CGI from three endoscopic images, the proposed network contains two dominant modules including the scaling feature fusion (SFF) module and section correlation (SC) module. Moreover, we also apply the channel attention (CA) layers to further extract attention features for better representations of different gastric sections. In the ablation study, we aim to evaluate the effectiveness of three schemes. The first three rows show the results of the proposed method with only the SFF module, CA layers and the SC module, respectively. The accuracy drops when only one of the proposed schemes is applied. Among these three schemes, the method with only the SFF module achieves better results. Such results indicate that it is necessary to generate multiple field-of-view features to overcome the problem that endoscopic images are captured under variant viewing angles and scales. Compared with the method with only the SC module, the method with only the CA layers achieves better sensitivity but has more false alarms. Because using only one scheme cannot achieve satisfactory results, collaborations of these schemes are applied.

As shown in the fourth row of Table II, the method without (w/o) using the SC module achieves the worst results when we combine different schemes. Without the SC module, the network is hard to build correlations among different gastric sections to represent CGI and thus has lower sensitivity. Such results indicate the importance and uniqueness of the proposed SC module based on the medical prior knowledge [15]. As shown in the fifth and sixth rows of Table II, our method without CA achieves better results compared with our method without the SFF module. Such results show that the proposed SFF module is more effective compared with the CA layers. The SFF module is proposed to capture the multiple field-of-view information of the endoscopic images. The proposed method without the SFF module was implemented by considering only the single field-of-view information, i.e. the large scale deep features of each endoscopic image. As shown in Table II, the proposed method without the SFF module achieves worse results compared with the proposed method. Thus, representing the mucosa under variant viewing directions and scale changes during endoscopy by using the SFF module is necessary.

**TABLE II:** Ablation Study

SFF	CA	SC	Acc.	Sens.	Spec.	AUC
√			0.8297	0.8750	0.8205	0.9326
	√		0.8085	0.9375	0.7820	0.9150
		√	0.8085	0.6875	0.8333	0.8645
√	√		0.9043	0.6875	0.9487	0.9647
√		√	0.9362	0.8750	0.9487	0.9792
	√	√	0.9255	0.7500	0.9615	0.9639
√	√	$\surd *$	0.9362	0.6875	0.9872	0.9655
√	√	√	**0.9574**	**0.9375**	**0.9615**	**0.9836**

In our method, we propose three section correlation losses. The first section correlation loss explains the medical prior knowledge of the correlations between the antrum and body, while the second section correlation loss explains the medical prior knowledge of the correlations between the antrum and cardia. The third section correlation loss $\ell ^{ABC}$ aims to provide additional correlations among the antrum, body and cardia in the SC module. The seventh row of Table II shows the results without considering the $\ell ^{ABC}$. Without $\ell ^{ABC}$, the network achieves worse results compared with the proposed method shown in the eighth row of Table II. Thus, applying $\ell ^{ABC}$ is necessary to the CGI diagnosis. These results show the effectiveness of the combinations of the proposed modules, channel attention layer, and section correlation losses for the CGI diagnosis.

### C. Quantitative Results

To the best of our knowledge, no existing artificial intelligence (AI) methods are proposed to diagnose CGI. The proposed method is the first AI method to solve the CGI diagnosis problem by using endoscopic images. Current deep learning methods are also hard to discover the correlations of different gastric sections, because they generally consider to learn features from images of a specific gastric section. For comparisons, we modify the input of the state-of-the-art convolutional neural network methods including GoogLeNet [30], ResNet-50 [27] and DenseNet-121 [31], and transformer based methods including BoTNet [32] and vision transformer (ViT) [33], so these methods can also learn deep features of different gastric sections for the CGI diagnosis. We concatenate endoscopic images of each patient's antrum, body, and cardia to a 9 channel image and pass the image to a $1 \times 1$ convolutional layer to learn a 3 channel image which serves as the input of these deep learning methods. The pre-trained models of the competing methods were loaded as the initial models to train the endoscopic images. In this way, these methods can learn better features of training endoscopic images based on the pre-trained models for fair comparisons in the CGI diagnosis.

The quantitative results are shown in Table III. Because the state-of-the-art methods are not designed for solving the CGI diagnosis problem, their results are not satisfactory. It is worth to note that the patch embeddings in ViT consider patches of a fixed field-of-view. Moreover, the position embeddings in ViT do not model multiple field-of-view information. Thus, ViT is hard to properly learn features of the endoscopic images captured under significant image scale changes and viewing angle changes. As a result, the performance of ViT is not satisfactory. In contrast, the proposed method outperforms all of the competing methods. Such results also indicate the difficulty of the CGI diagnosis when the correlations of three gastric sections are not correctly represented. While the scaling feature fusion module helps learn representative features of each gastric section under variant viewing angles and scales, the channel attention layers help extract more salient features. Then, the section correlation module fuses the learned features based on the medical prior knowledge in [15], i.e. the correlations of different gastric sections, and makes our method become more interpretable with three proposed section correlation losses. As a result, our network can successfully diagnose CGI from the patients' endoscopic images without invasive biopsies and time-consuming process of the updated Sydney system which burdens the pathologists in [15]. In addition, the average inference time during testing is 0.0253 s which shows the real-time processing ability of the proposed method for online endoscopy.

**TABLE III:** Quantitative Results

Method	Acc.	Sens.	Spec.	AUC
GoogLeNet	0.8723	0.6875	0.9103	0.8726
ResNet-50	0.8936	0.6875	0.9487	0.9195
DenseNet-121	0.8408	0.7500	0.8590	0.8766
BoTNet	0.8617	0.8125	0.8718	0.8950
ViT	0.7979	0.7500	0.8077	0.8910
Proposed	**0.9574**	**0.9375**	**0.9615**	**0.9836**

### D. Qualitative Results

**Fig. 2.**
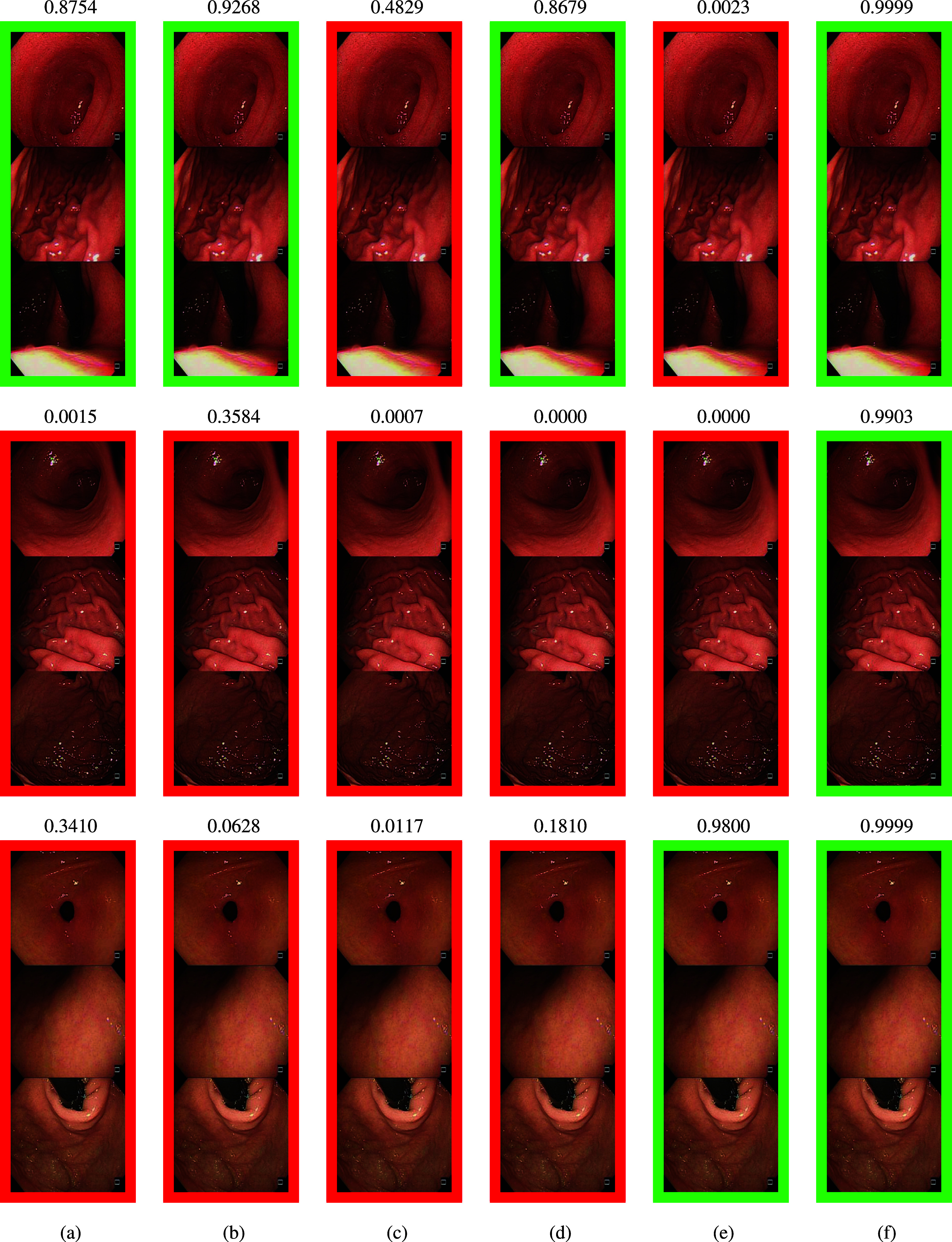
The qualitative results of the state-of-the-art methods and the proposed method. The confidence probability of each method is shown above the result for (a) GoogLeNet, (b) ResNet, (c) DenseNet-121, (d) BoTNet, (e) ViT, and (f) Proposed method.

Fig. 2 shows the CGI diagnosis results of the state-of-the-art methods and the proposed method for three patients, where the first two patients are diagnosed as CGI and the third patient is diagnosed as normal by the pathologists. We group the endoscopic images of the antrum, body and cardia of each patient from the top to the bottom for visualization. As shown in Fig. 2, the variations of appearances of mucosa and the viewing angles of the endoscopic cameras are significantly different. These variations lead to the difficulty of the CGI diagnosis. Moreover, CGI is diagnosed based on the correlations of inflammations of different gastric sections [15]. Thus, the endoscopists are hard to manually review and compare the correlations of inflammations of different sections during endoscopy for the CGI diagnosis, because the camera can only capture a gastric section in each view.

Fig. 2(a), (b), (c), (d), and (e) show the results of conventional deep learning methods of GoogLeNet, ResNet-50, DenseNet-121, BoTNet and vision transformer, respectively. The green and red rectangles represent the correct and incorrect CGI diagnosis results of each patient, respectively. The confidence probability of each method is shown above the results. Because these methods are hard to learn the proper correlations of three different gastric sections, they fail to correctly diagnose CGI due to variant camera viewing angles and scale changes. As shown in the third row of Fig. 2, GoogLeNet, ResNet, DenseNet-121 and ViT also miss-classify the normal patient to the patient with CGI. The results of the proposed shown are shown in Fig. 2(f). While the scaling feature fusion module learns robust deep features to overcome the viewing angle and scale changes, the section correlation module employs the medical prior knowledge to learn the correlations between different gastric sections for the CGI diagnosis. As a result, the proposed method can achieve significantly better results with high confidence probability compared with the competing methods

## IV. Conclusion

The early diagnosis of the gastric precancerous lesion, CGI, helps reduce the mortality rate and occurrence of gastric cancer. However, the current CGI diagnosis can only be achieved by manual review from invasive biopsy specimens, and is a very time-consuming and burden process for pathologists and physicians. In this paper, we propose a novel gastric section correlation network which is the first artificial intelligence method to achieve the CGI diagnosis. The scaling feature fusion module aims to represent the scale and viewing angle changes during endoscopy, while the section correlation module applies the medical prior knowledge to achieve better CGI diagnosis results. Applying the medical prior knowledge not only makes our method become more interpretable but also helps solve the CGI diagnosis problem for the purposes of the precision medicine and preventive healthcare.

The experimental results show that the proposed method significantly outperforms all of the state-of-the-art methods. Our method takes into account the medical prior knowledge to build correlations of endoscopic images of different gastric sections, while existing deep neural network models only consider content of individual endoscopic images. We show that the CGI diagnosis from endoscopic images can be achieved, and thus the invasive biopsy and time-consuming process of the conventional CGI diagnosis can be avoided. In the future, we will append the proposed model to the video endoscopy system for the online CGI diagnosis.
